# Case Report: Extra-adrenal retroperitoneal paraganglioma in a young adult cat diagnosed by imaging, pathology, and immunohistochemistry

**DOI:** 10.3389/fvets.2025.1636725

**Published:** 2025-08-26

**Authors:** Sang-June Sohn, Sohee Lim, Junghoon Park, Ulsoo Choi, Yeon-Jung Hong

**Affiliations:** ^1^Western Referral Animal Medical Center, Seoul, Republic of Korea; ^2^College of Veterinary Medicine, Jeonbuk National University, Iksan, Republic of Korea

**Keywords:** paraganglioma, extra-adrenal, pheochromocytoma, neuroendocrine tumor, retroperitoneal mass, immunohistochemistry, cat

## Abstract

Paragangliomas are rare neuroendocrine tumors arising from paraganglionic tissues associated with the autonomic nervous system. Although they are uncommon in veterinary medicine, particularly in cats, accurate diagnosis is essential due to their potential malignancy. An 18-month-old neutered male domestic shorthair cat presented with a retroperitoneal mass near adrenal glands. Computed tomography revealed an irregular, mildly contrast-enhancing mass abutting both adrenal glands and displacing adjacent vessels. Fine-needle aspiration cytology suggested a malignant round cell tumor with differential diagnosis of lymphoma, nephroblastoma, or primary embryonal tumor. Surgical excision was performed and histopathological examination identified a high-grade round cell neoplasm with a high mitotic index. A broad panel of immunohistochemistry excluded lymphoma and nephroblastoma, confirming extra-adrenal paraganglioma based on strong positivity for anti-synaptophysin, neuron-specific enolase, chromogranin A, and cytokeratin 19 antibodies. On postoperative day two, the cat developed suspected cranial mesenteric artery thrombosis leading to mesenteric ischemia and eventually euthanasia due to poor prognosis. This case emphasizes the importance of considering extra-adrenal paraganglioma in differential diagnoses of retroperitoneal masses in young cats and highlights the critical role of advanced imaging and immunohistochemistry in achieving definitive diagnosis.

## 1 Introduction

Paragangliomas are rare neuroendocrine tumors that originate from paraganglionic tissues associated with the autonomic nervous system. These neoplasms arise from neural crest-derived chromaffin cells. Based on their locations, they are classified as either adrenal (pheochromocytomas) or extra-adrenal paragangliomas (EAPs) according to the World Health Organization (1). Pheochromocytomas are typically confined to the adrenal medulla. They often secrete catecholamines. EAPs arise from either sympathetic or parasympathetic ganglia and exhibit variable secretory activities. Sympathetic EAPs often located along the paravertebral axis are more likely to be functional, whereas parasympathetic EAPs typically found in the head and neck region are generally non-functional with a higher risk of malignancy. The clinical diagnosis of paragangliomas can be challenging due to their nonspecific clinical signs and the absence of pathognomonic imaging features, which necessitate the use of advanced diagnostic modalities (2–5). In veterinary medicine, ultrasonography and computed tomography (CT) play an important role in the detection, anatomical localization, and characterization of retroperitoneal masses, and can lead to incidental identification of clinically silent tumors (6, 7). Although paragangliomas have a low incidence ranging from 0.04 to 0.95 cases per 100,000 people per year in humans (8), they are even rarer in veterinary medicine, with most reports involving dogs (6). In cats, only sporadic cases of paragangliomas have been reported in locations such as the cauda equina (9), kidneys (10), orbit (11), heart (12), and retroperitoneum (7, 13, 14). To the authors’ knowledge, this report describes the youngest feline case of retroperitoneal EAP reported to date (7, 9–14). Furthermore, immunohistochemistry (IHC) in this case revealed cytokeratin 19 (CK19) expression, a finding not previously documented in veterinary paragangliomas, highlighting the potential diagnostic and biological significance of immunohistochemical profiling in these tumors.

Here, we describe a rare case of retroperitoneal EAP in a young adult cat. It was definitively diagnosed via histopathology and immunohistochemistry (IHC) following advanced diagnostic imaging.

## 2 Case description

**Figure 1:**
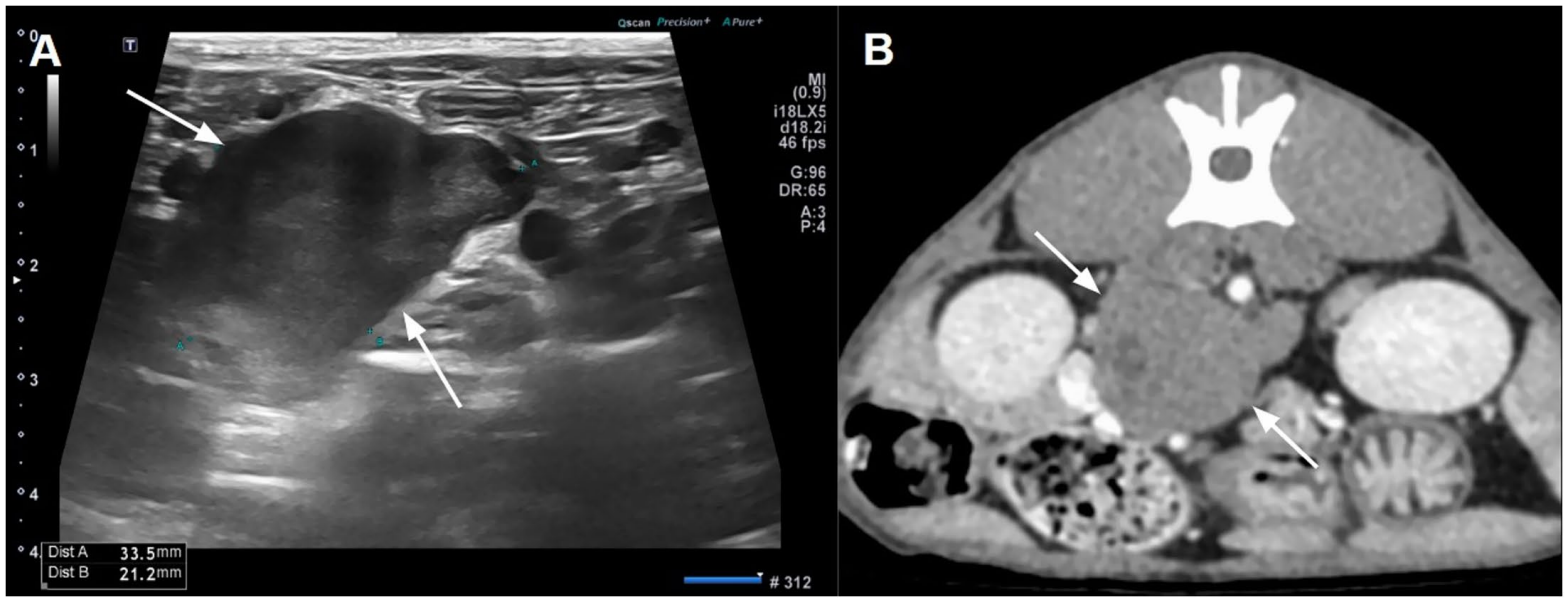
Abdominal ultrasonography **(A)** and computed tomography **(B)** illustrating a hypoechoic and mild contrast-enhancing mass between both adrenal glands. A small hypodense area reflecting cystic degeneration was shown in the mass.

A 1-year-old, 3.9 kg, male neutered domestic shorthair cat was referred to Western Animal Center for further evaluation after ultrasonography at the referring clinic incidentally revealed a heterogeneous hypoechoic mass near the right adrenal gland. On physical examination, blood pressure was elevated at approximately 180 mmHg, suggesting the possibility of white coat effect or pathological hypertension. A complete blood count and serum chemistry revealed no significant abnormalities except for mildly elevated alkaline phosphatase activity (202 U/L; reference range, 38–165 U/L) and phosphate (6.4 mg/dL; reference range, 2.6–6 mg/dL). Thoracic radiographs provided by the referring hospital revealed no remarkable findings. On ultrasonography, a mildly heterogeneous, hypoechoic mass was observed in close proximity to both adrenal glands (Figure 1A). A computed tomography (CT) scan was performed to determine the origin of the mass and to aid in surgical planning.

**Figure 2:**
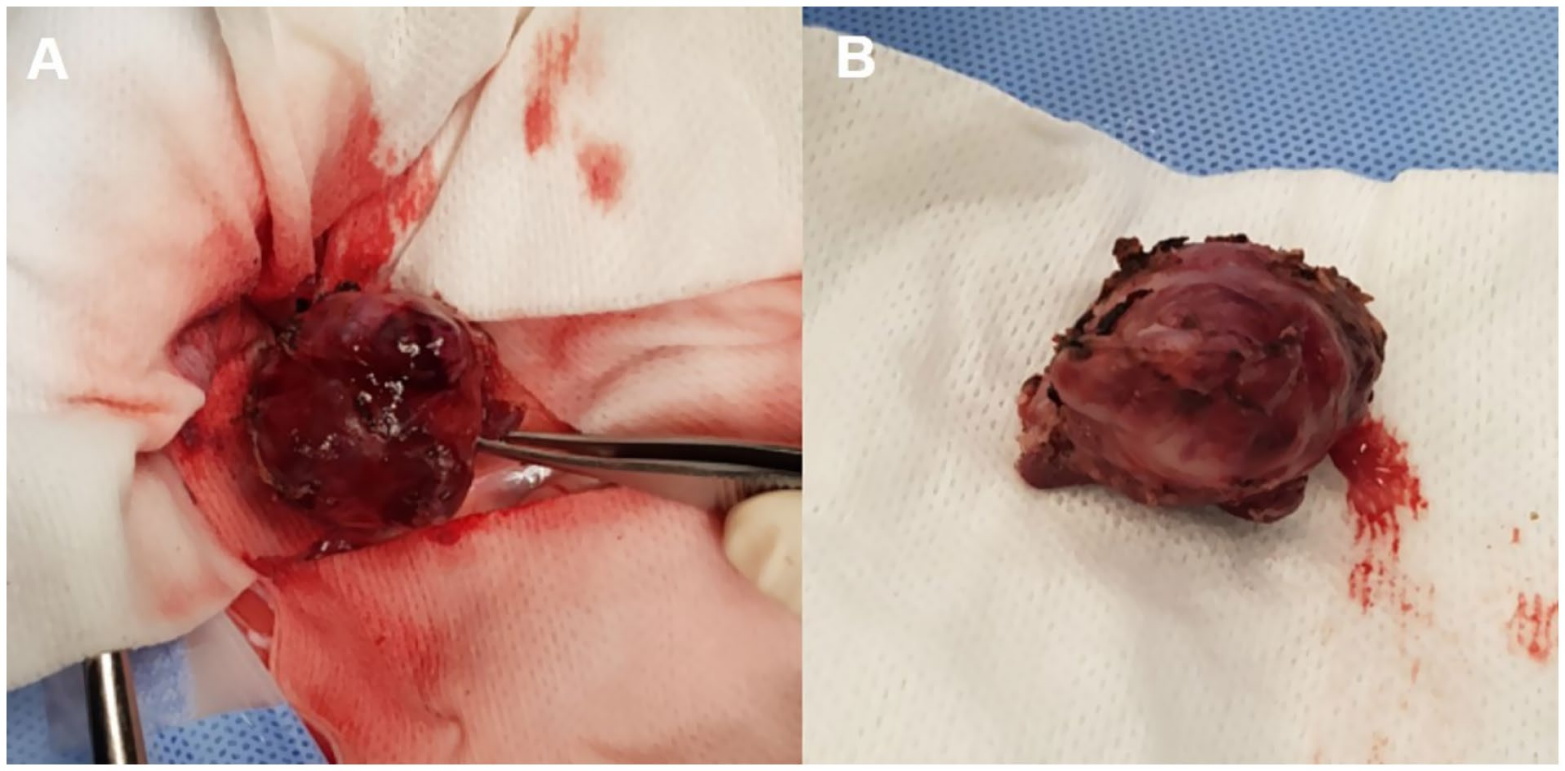
Preoperative images revealing an irregular oval-shaped mass adjacent to both adrenal glands.

Abdominal CT revealed an irregular, soft tissue attenuated, retroperitoneal mass (3 × 2.5 × 3.5 cm) closely abutting both adrenal glands (Figure 2B). The mass demonstrated mild contrast enhancement (approximately 60–70 HU) compared to adrenal glands (about 150 HU). It showed a non-enhancing intracystic structure with compression and displacement of adjacent caudal vena cava and portal vessels. Mild lymphadenopathy of jejunal, hepatic, and splenic lymph nodes was observed, which was considered a reactive lymphadenopathy. Thoracic CT showed no significant abnormalities. Fine-needle aspiration (FNA) cytology demonstrated a cellular population of large round cells with a high nuclear-to-cytoplasmic ratio, scant basophilic cytoplasm, prominent nucleoli, and frequent mitotic figures suggesting malignant round cell neoplasia. Differentials included high-grade lymphoma, primary embryonal tumors, and other undifferentiated neoplasms.

**Figure 3:**
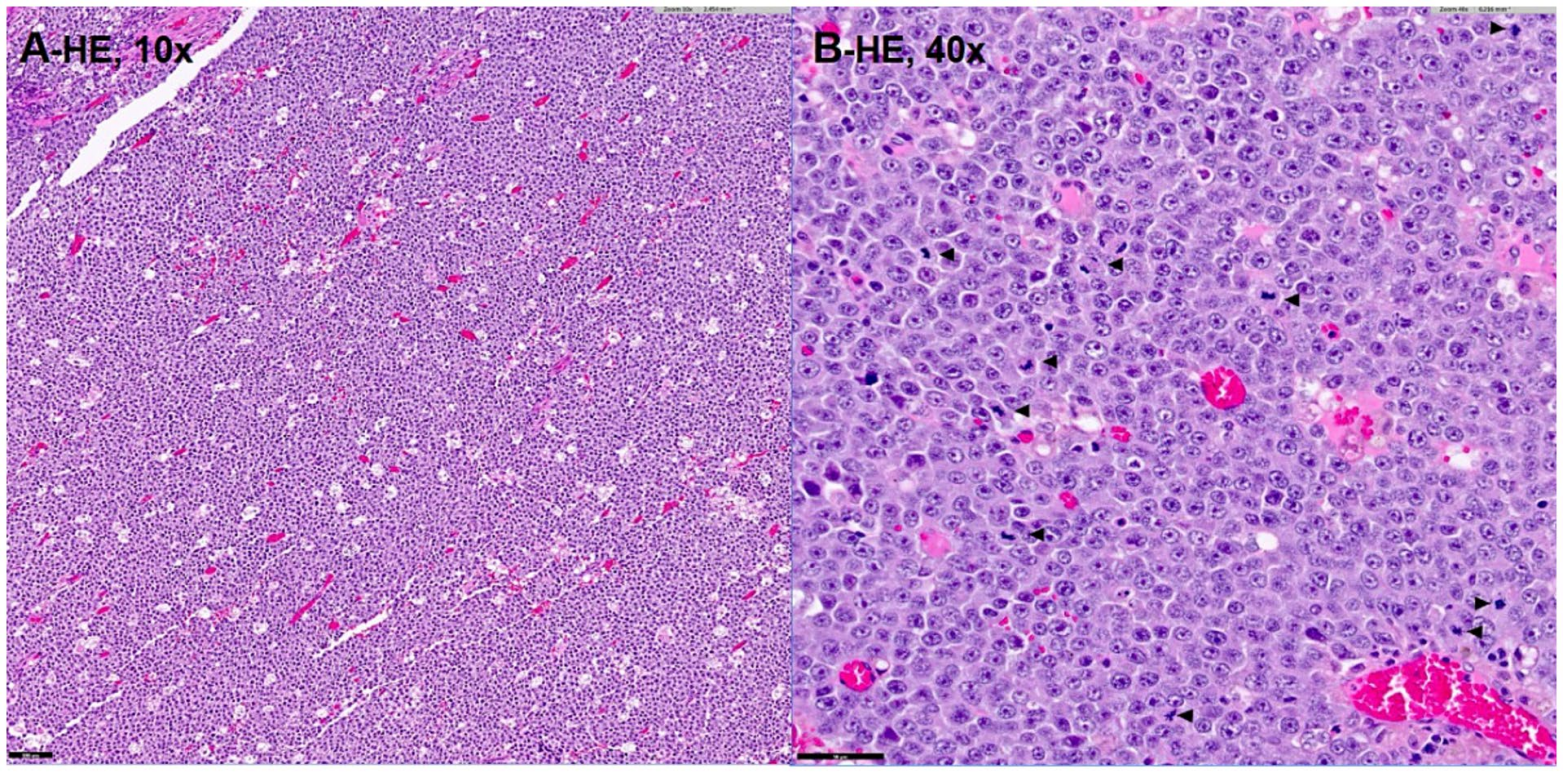
Histopathologic image **(A,B)** demonstrating a densely cellular, high-grade large cell round cell neoplasm with marked nuclear atypia and a high mitotic index (**B**; arrowhead). Hematoxylin–Eosin stain, magnification of 10x **(A)**, 40x **(B)**. Bar = 100 μm in **(A)** and 50 μm in **(B)**.

**Figure 4:**
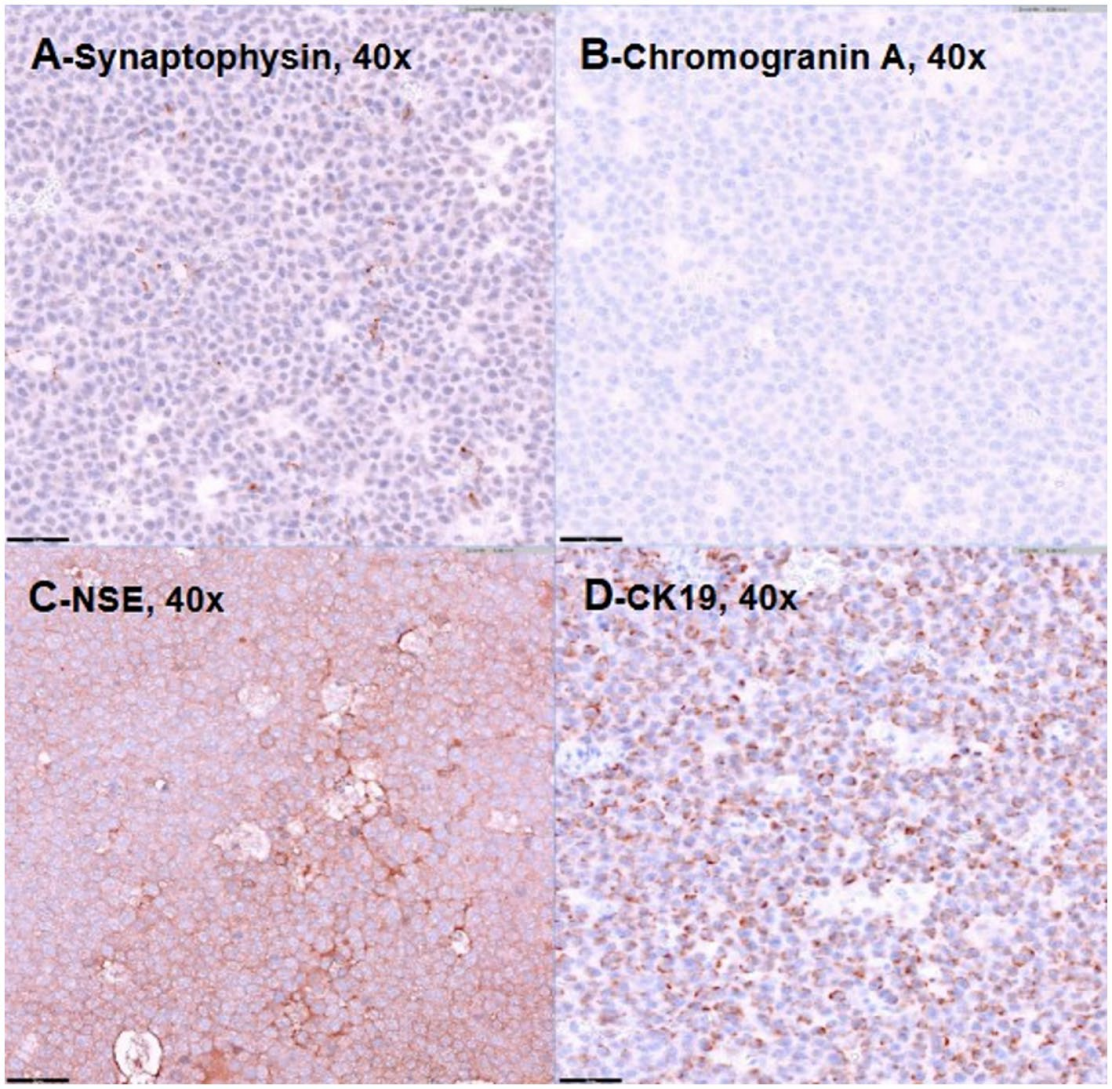
Representative images demonstrating positive immunostaining for synaptophysin **(A)**, chromogranin-A **(B)**, neuron-specific enolase **(C)**, and CK19 **(D)**. Bar = 50 μm.

The irregular and oval-shaped mass was surgically excised without significant postoperative hemorrhage (Figure 2). Dissection from the renal vein and caudal vena cava was achieved at the dorsolateral aspect. The mass was removed en bloc with its capsule except at regions adjacent to both adrenal glands where excision was performed without the capsule. Histopathologic examination revealed a densely cellular, non-encapsulated, large monomorphic round cell tumor with marked nuclear atypia and a high mitotic index (46 per 10 HPFs per 2.37 mm^2^; Figures 3A,B). Neoplastic cells were monomorphic and arranged in sheets and aggregates, displaying distinct cell borders, scant eosinophilic cytoplasm, and large round nuclei with finely stippled chromatin and prominent nucleoli. Marked anisocytosis and anisokaryosis were observed with nuclei exceeding twice the size of erythrocytes. These features were initially consistent with a high-grade lymphoma exhibiting aggressive biologic behavior. The mass was completely excised with narrow surgical margins (< 0.1 mm). Initial IHC results excluded lymphoma and nephroblastoma as neoplastic cells lacked expression of CD3 and PAX5 for lymphoma and Wilms’ tumor 1 for nephroblastoma. Given the tumor’s anatomical location and histomorphologic features, paraganglioma emerged as the leading differential diagnosis. Subsequent immunohistochemical analysis revealed strong, diffuse positivity for neuron-specific enolase (NSE) and synaptophysin, with chromogranin A positivity in 10–20% of cells, supporting a neuroendocrine origin (Figures 4A–C). CK19 expression was detected in up to 40% of neoplastic cells in a punctate cytoplasmic pattern (Figure 4D), while vimentin was weakly expressed (5%). Negative staining for neurofilament and desmin effectively ruled out neuronal and myogenic differentiation. The mass was finally diagnosed as retroperitoneal EAP.

On the second day of hospitalization, abdominal ultrasonography revealed mesenteric edema at the surgical site and an absence of blood flow on color Doppler within part of the cranial mesenteric artery, raising suspicion for acute mesenteric infarction. On the third day, echogenic peritoneal effusion was significantly increased and generalized small intestinal wall edema was more pronounced than previously noted. In the duodenal region, loss of mural layering, irregular wall thickening, and possible disruption of wall continuity were observed, raising concern for intestinal leakage. Because of a grave prognosis, euthanasia was elected by the owner on the fourth day of hospitalization.

## 3 Discussion

This report describes a rare case of malignant retroperitoneal EAP confirmed through advanced imaging, pathology, and IHC in a domestic cat. Paragangliomas are rare neuroendocrine tumors and infrequently encountered in veterinary medicine, particularly in cats. Among previously reported retroperitoneal cases, affected cats were 7–18 years old (7, 13, 14). Paragangliomas at other sites have also been described in old cats ranging from 5 to 18 years of age (9–12). This case involved an 18-month-old cat, representing the youngest reported feline case of EAP to date. This suggests that EAPs may occur across a wider age spectrum than previously recognized. Thus, EAPs should be considered as a differential diagnosis even in young cats. Clinical signs of veterinary EAPs are often nonspecific. They vary depending on tumor location, size, and functional status. Reported clinical signs in cats and dogs include lethargy, polydipsia, intermittent vomiting, anorexia, and depression (6, 7, 13, 14). In the present case, the retroperitoneal mass was discovered incidentally during abdominal ultrasonography as the patient exhibited no overt clinical signs. This supports the possibility that retroperitoneal EAPs may remain clinically silent, particularly when they are non-functional and located in anatomically accommodating regions.

Imaging findings in this case were partially consistent with previously reported features. On ultrasonography, retroperitoneal paragangliomas in dogs are typically hypoechoic relative to surrounding fat (6). In a feline case, a large retroperitoneal mass with a thick hyperechoic capsule and hypoechoic center has been described (7). In our case, the mass appeared mildly heterogeneous and hypoechoic without a distinct capsule. Compared with previous reports, the mass appeared relatively more uniform, possibly reflecting a less advanced disease stage or a lower degree of internal degeneration at the time of imaging. On CT, retroperitoneal paragangliomas in both humans and dogs are often characterized by intense contrast enhancement and heterogeneous internal architecture due to rich vascularization and frequent necrosis or cystic degeneration (2, 3, 6). A similar pattern was reported in a feline case (7), where the retroperitoneal mass displayed marked and heterogeneous capsular enhancement (up to 133 ± 9 HU) and a hypoattenuating non-enhancing center (23 ± 5 HU), consistent with necrotic or cystic degeneration. These imaging features, including displacement of adjacent organs and encasement of vascular structures, reflected a more advanced or biologically active lesion. In contrast, CT findings in the present case demonstrated a lobulated retroperitoneal mass with only mild contrast enhancement (60–70 HU), substantially lower than typical adrenal attenuation approximately 150 HU. CT also revealed a relatively uniform appearance, aside from small intralesional cystic areas. This milder enhancement pattern might reflect lower vascularity, limited internal necrosis, or an earlier stage of tumor development compared to previously documented feline and canine cases. These contrasting imaging characteristics illustrate the heterogeneity of retroperitoneal paragangliomas. They also reinforce an inherent diagnostic challenge due to the lack of pathognomonic imaging features. Retroperitoneal paragangliomas may share radiologic characteristics with other retroperitoneal neoplasms such as neurofibromas, neuromas, or soft tissue sarcomas, which can hinder accurate preoperative identification. In human medicine, CT-based preoperative misdiagnosis rates have historically reached up to 89% (3). Therefore, cross-sectional imaging findings must be interpreted with caution and always integrated with clinical, histopathologic, and immunohistochemical data to achieve a definitive diagnosis and guide appropriate treatment.

In the present case, the mass was initially suspected to be a high-grade lymphoma or another undifferentiated malignancy based on cytologic and histopathologic findings. However, IHC led to the final diagnosis of EAP. Positive staining for neuroendocrine markers (NSE, synaptophysin, chromogranin A) and negative staining for lymphoid (CD3, Pax5) and nephroblastoma (WT1) markers were pivotal in confirming the tumor’s neuroendocrine origin. Based on histologic appearance alone, the primary differential diagnoses included high-grade lymphoma, nephroblastoma, and neuroblastoma. Lymphoma was suspected due to the monomorphic round cell morphology, large nuclei, and high mitotic activity. However, IHC showed complete negativity for lymphoid markers (CD3 and Pax5). Cytologically, the neoplastic cells were large and round with prominent nucleoli and scant cytoplasm, lacking granules or vacuoles typically seen in neuroblastic or nephrogenic tumors. While imaging findings were not pathognomonic, the mass’s anatomical position and relatively uniform enhancement pattern were considered less typical for nephroblastoma (2). Nephroblastoma and neuroblastoma were further ruled out by negative WT1 and neurofilament staining, respectively. The absence of tubular or primitive glomeruloid structures on histopathology additionally argued against nephroblastoma. This case supports the importance of a multimodal diagnostic approach that integrates advanced imaging, histopathology, and IHC for accurate diagnosis of rare retroperitoneal masses. A similar diagnostic challenge was reported in a case of feline renal paraganglioma, where histopathology alone suggested carcinoma or undifferentiated neuroendocrine neoplasia, but a definitive diagnosis was achieved following IHC and transmission electron microscopy (10). Taken together, these cases emphasize limitations of routine histopathology and the necessity of comprehensive diagnostic workups in rare feline neuroendocrine tumors. Interestingly, CK19 expression was observed in the present case, which has not been previously reported in veterinary paragangliomas to the authors’ knowledge. In human medicine, CK19 is widely expressed in gastrointestinal and hepatopancreatic neoplasms. It has been studied as a prognostic biomarker in pancreatic neuroendocrine tumors (PanNETs). CK19-positive PanNETs have been associated with larger tumor sizes, higher WHO grades, advanced local invasion, and increased rates of metastasis and lymphovascular invasion (15). These tumors are often correlated with more aggressive biological behavior and poorer clinical outcomes (16). Although the prognostic value of CK19 in veterinary paragangliomas remains unclear, its expression observed in the present case raises the possibility that CK19 might similarly reflect aggressive tumor biology in feline neuroendocrine neoplasms. However, given the single-case nature of this report and absence of follow-up data, such interpretations should be made with caution. CK19 expression may reflect partial epithelial differentiation, as reported in some human neuroendocrine tumors (16). Alternatively, nonspecific cross-reactivity cannot be excluded, particularly in the context of veterinary IHC. Further studies are needed to clarify the biological relevance of CK19 in veterinary oncology.

Despite complete surgical excision, the prognosis for retroperitoneal paragangliomas remains guarded. Surgical resection is the primary approach for both diagnosis and treatment in human and veterinary medicine. In humans, tumor removal has been shown to significantly improve survival outcomes. In one study, complete resection yielded 5- and 10-year disease-free survival rates of 75 and 45%, respectively, compared to 19% when tumors were not removed (5). Once metastasis occurred, 5-year survival dropped to 36%, with no patients surviving beyond 76 months. More recently, one study demonstrated a 5-year survival rate of 91% (3), although recurrence and metastasis remained high at 41.9%, with a mortality rate of 19% over an average follow-up of 51 months (4).

In veterinary medicine, long-term outcome data are limited. Among three reported feline cases of retroperitoneal EAPs, two cats were euthanized shortly after diagnosis or surgery (7, 13), while one cat remained clinically stable for 14 months postoperatively (14). A retrospective study of 10 canine cases revealed variable outcomes: one dog underwent successful excision with normalization of blood pressure, one was followed up for 2 years with supportive care, one died from an unrelated cause, and one was euthanized after CT evaluation (6). These findings underscore the variability in prognosis and reinforce the need for surgical excision, cautious and long-term monitoring, and further research into prognostic indicators in veterinary patients.

In the present case, despite grossly complete excision and no overt evidence of metastasis, the cat developed acute vascular complications suspected to be mesenteric infarction. Although mesenteric infarction is rare in veterinary species, it is a life-threatening condition characterized by sudden compromise of intestinal blood flow, leading to ischemia, necrosis, and often rapid clinical deterioration (17, 18). In human medicine, it is most commonly associated with hypercoagulable states, vascular embolism, or mass-related vascular compression with a high mortality rate. Although rare, acute mesenteric ischemia has also been described in dogs and cats with underlying trauma or cardiac disease (19, 20).

This case has several limitations, including the absence of long-term follow-up due to euthanasia shortly after surgery. This precludes assessment of recurrence or survival and limits interpretation of CK19’s prognostic significance.

In summary, this case expands the known age range of feline retroperitoneal EAPs, illustrates the diagnostic challenge posed by their nonspecific clinical and imaging features, and demonstrates the value of multimodal diagnostics, including IHC, in achieving a definitive diagnosis. CK19 expression, while novel, should be interpreted cautiously until further veterinary studies are available. This case also highlights the importance of considering EAP in the differential diagnosis of retroperitoneal masses in cats of any age and the necessity for careful postoperative monitoring given the risk of acute vascular events and uncertain prognosis.

## 4 Conclusion

This report describes a rare case of extra-adrenal retroperitoneal EAP in a young adult cat. The EAP was definitively diagnosed by histopathology and IHC. This case highlights the importance of considering neuroendocrine neoplasms as differentials for retroperitoneal masses in cats regardless of age. It also reinforces the diagnostic value of advanced imaging and immunophenotyping in characterizing poorly differentiated neoplasms. While rare, feline retroperitoneal EAPs may present with non-specific clinical signs. They warrant comprehensive investigation to reach a definitive diagnosis.

## Data Availability

The original contributions presented in the study are included in the article/supplementary material, further inquiries can be directed to the corresponding authors.
